# Cerebellum/liver index on baseline 18F-FDG PET/CT to improve prognostication in post-transplant lymphoproliferative disorders: a multicenter retrospective study

**DOI:** 10.1186/s13550-024-01111-8

**Published:** 2024-05-27

**Authors:** David Morland, Lukshe Kanagaratnam, Fabrice Hubelé, Elise Toussaint, Sylvain Choquet, Aurélie Kas, Pierre-Ambroise Caquot, Corinne Haioun, Emmanuel Itti, Stéphane Leprêtre, Pierre Decazes, Fontanet Bijou, Paul Schwartz, Caroline Jacquet, Adrien Chauchet, Julien Matuszak, Nassim Kamar, Pierre Payoux, K-VIROGREF Study Group, Loïc Renaud, Laetitia Vercellino, Jérôme Paillassa, Pacôme Fosse, Morgane Cheminant, Jean Michel Correas, Roch Houot, Xavier Palard, Marie Le Cann, Maria-Angéla Castilla-Lièvre, Yann Guillermin, Haifa Bahri, Eric Durot

**Affiliations:** 1 Médecine Nucléaire, Institut Godinot, Reims, France; 2 Laboratoire de Biophysique, UFR de Médecine, Université de Reims Champagne-Ardenne, Reims, France; 3 CReSTIC, EA 3804, Université de Reims Champagne-Ardenne, Reims, France; 4 Unità di Medicina Nucleare, TracerGLab, Dipartimento di Radiologia, Radioterapia ed Ematologia, Fondazione Policlinico Universitario A. Gemelli, IRCCS, Rome, Italy; 5 Unité d’Aide Méthodologique, Pôle Recherche et Santé Publique, CHU de Reims, Reims, France; 6 Médecine Nucléaire, CHU de Strasbourg, ICANS, Strasbourg, France; 7 Hématologie, CHU de Strasbourg, ICANS, Strasbourg, France; 8 Hématologie, CHU Pitié-Salpêtrière Charles Foix, Sorbonne Université, AP-HP, Paris, France; 9 Médecine Nucléaire, CHU Pitié-Salpêtrière Charles Foix, Sorbonne Université, AP-HP, Paris, France; 10 Hématologie, CHU Henri Mondor, AP-HP, Créteil, France; 11 Médecine Nucléaire, CHU Henri Mondor, AP-HP, Créteil, France; 12 Inserm U1245 et Département d’Hématologie, Centre Henri Becquerel et Normandie Univ, UNIROUEN, Rouen, France; 13 Médecine Nucléaire, Centre Henri Becquerel, Rouen, France; 14 Hématologie, Institut Bergonié, Bordeaux, France; 15 Médecine Nucléaire, Institut Bergonié, Bordeaux, France; 16 Hématologie, CHU de Nancy, Nancy, France; 17 Hématologie, CHU de Besançon, Besançon, France; 18 Médecine Nucléaire, CHU de Besançon, Besançon, France; 19 Néphrologie et transplantation d’organes, CHU Rangueil, Toulouse, France; 20 Médecine Nucléaire, CHU de Toulouse, Toulouse, France; 21 Hématologie Clinique, CHU de Reims, Reims, France; 22 Hématologie, Hôpital Saint-Louis, APHP, Paris, France; 23 Médecine Nucléaire, Hôpital Saint Louis, APHP, Paris, France; 24 Hématologie, CHU d’Angers, Angers, France; 25 Médecine Nucléaire, CHU d’Angers, Angers, France; 26 Hématologie, Hôpital Necker, APHP, Paris, France; 27 Radiologie Adulte, Hôpital Necker, Paris, France; 28 Hématologie, CHU Rennes, Rennes, France; 29 Médecine Nucléaire, Centre Eugène Marquis, Rennes, France; 30 Hématologie, Hôpital Bicêtre, APHP, Le Kremlin-Bicêtre, France; 31 Médecine Nucléaire, Hôpital Antoine Béclère, APHP, Clamart, France; 32 Hématologie, Centre Léon Bérard, Lyon, France; 33 Médecine Nucléaire, Centre Léon Bérard, Lyon, France

**Keywords:** Lymphoma, Immunocompromised host, Cerebellum, Fluorodeoxyglucose F18, Positron-emission tomography

## Abstract

**Background:**

Besides International Prognostic Index (IPI) score, baseline prognostic factors of post-transplant lymphoproliferative disorders (PTLD) are poorly identified due to the rarity of the disease. New indexes derived from healthy organ uptake in baseline 18F-FDG PET/CT have been studied in immunocompetent lymphoma patients. The aim of this study is to evaluate the performances of the cerebellum-to-liver uptake ratio (denoted as CLIP) as a prognostic factor for PFS and OS. This retrospective multicenter study is based on patients with PTLD included in the K-VIROGREF cohort. The previously published threshold of 3.24 was used for CLIP in these analyses.

**Results:**

A total of 97 patients was included with a majority of monomorphic diffuse large B-cell lymphoma subtype (78.3%). Both IPI score (≥ 3) and CLIP (< 3.24) were significant risk factors of PFS with corresponding hazard ratios of 2.0 (1.0–4.0) and 2.4 (1.3–4.5) respectively. For OS, CLIP was not significant and resulted in a hazard ratio of 2.6 (*p* = 0.059). Neither IPI score or Total Metabolic Tumor Volume reached significance for OS.

**Conclusion:**

CLIP is a promising predictor of PFS and perhaps OS in PTLD. Further prospective studies are needed to confirm these results.

## Introduction

About 130,000 patients received solid organ transplants in 2020. Cumulatively, 1–9% of transplant recipients are affected by post-transplant lymphoproliferative disorders (PTLD) [1, 2]. The prognostic factors of PTLD are poorly known due to the rarity of this disease. The International Prognostic Index (IPI) score is described in the few available articles, as well as response to rituximab induction when this regimen is used [1, 3, 4].

FDG PET is inconsistently used in PTLD but showed excellent sensitivity (90.0%) and specificity (90.0%) for lesion detection [5, 6]. However, in contrast with other FDG-avid lymphomas in immunocompetent patients, baseline Total Metabolic Tumor Volume (TMTV) and Total Lesion Glycolysis were not predictive of overall survival in PTLD [4].

New indexes based on healthy organ uptake have been successfully used in a few lymphoma publications [7, 8]. These indices are based on the “tumor sink effect” principle, which has also been described with other radiopharmaceuticals [9]: a more aggressive and/or larger tumor is supposed to capture the tracer at the expense of healthy organs whose uptake decreases. Cerebellar uptake, divided by hepatic uptake for normalization purposes, has been suggested as a potential prognostic factor in diffuse large B-cell lymphoma [8] and follicular lymphoma [7] in immunocompetent patients.

The main objective of this study is to evaluate the potential of the cerebellum/liver index (denoted as CLIP: cerebellum/liver index for prognosis) to predict Progression Free Survival (PFS) and Overall Survival (OS) in PTLD using the threshold previously reported [8]. As a secondary exploratory objective, we will study the value of this index in relation to the other parameters usually studied in FDG PET/CT by recalculating the thresholds for our study population (TMTV, Total Lesion Glycolysis, SUVmax, ratios with hepatic or blood pool uptake).

## Material and methods

### Patients’ selection

This retrospective, non-interventional, multicenter study is based on patients included in the K-VIROGREF cohort (epidemiological, clinical, and immunological study of a cohort of adult patients with viral-induced cancers, after solid organ and hematopoietic stem cell transplantation). Patients with PTLD were screened from July 2013 to October 2021. Inclusion criteria were as follow: histologically proven polymorphic or monomorphic PTLD; available baseline 18F-FDG PET/CT, performed within 30 days prior treatment. Exclusion criteria were: indolent lymphomas; previously treated PTLD; Central nervous system involvement; noncompliance with fasting prior to PET; incomplete DICOM data.

The diagnosis of PTLD was made in accordance with the WHO classification [10] of malignant lymphoma and confirmed by expert hematopathologists from the Lymphopath network, according to the standard French procedures.

### Data collection

For each patient, the following parameters were collected from the K-VIROGREF registry: (1) clinical data including age at diagnosis, sex, Eastern Cooperative Oncology Group Performans Status (ECOG PS), B symptoms; (2) transplantation related data: time between transplantation and diagnosis of PTLD, age at transplantation, transplanted organ; (3) lymphoma characteristics: histology, Epstein Barr Virus status of the tumor (EBER), Ann Arbor stage, Nodal involvement, extranodal involvement, graft involvement; (4) International Prognostic Index (IPI), LDH, Bêta-2 microglobulin and albumin levels; (5) Treatment strategy, including reduction of immunosuppression. PFS was calculated from diagnosis until disease progression, relapse or death from any cause or last follow-up. OS was defined from diagnosis to death or last follow-up.

Regarding 18F-FDG PET/CT: DICOM data and weight were collected.

### Baseline PET measurements

PET/CT were displayed on a dedicated interpretation console (AW server, General Electrics, USA). Cerebellum/Liver index was measured as previously described [7, 8]. The SUVmax of the cerebellum was measured using an enclosing region of interest (ROI) excluding any voxel of the neighboring brain hemispheres. A default cubic ROI of 72 cm^3^ (41% SUVmax threshold) was positioned in the right liver to measure its SUVmean. CLIP is the ratio of the SUVmax of the cerebellum divided by the SUVmean of the liver. This measurement technique has been proven to be reproducible and not dependent on the type of region of interest used (thresholding or not, cubic or spherical shape) [7, 8].

Total metabolic tumor volume (TMTV) and total lesion glycolysis (TLG) were obtained by summing the metabolic volumes of all nodal and extranodal lesions according to the method detailed by Meignan et al. [11] (41% SUVmax threshold, inclusion of only focal bone marrow involvement, spleen considered involved in case of focal increased uptake or diffuse increased uptake of at least 1.5 times the liver uptake).

For exploratory purposes, other parameters were measured: the SUVmax of the lymphoma lesion with the greatest uptake was collected, as was the SUVmean of the lymphoma (TLG/TMTV); SUVmean blood pool was measured using a spherical region of interest placed in the aorta. Commonly used ratios (SUVmax tumor/SUVmean liver and SUVmax tumor/SUVmean bloodpool) were calculated.

All measurements were performed by an experienced nuclear medicine physician (DM) who was blinded to the clinical data of the patients.

### Statistical analysis

For descriptive analysis, qualitative variables were described by their absolute and relative frequency (%). Quantitative variables were described by mean, standard deviation. Median, interquartile range (IQR) and extreme values are provided in addition for TMTV. Comparisons between patients with CLIP < 3.24 and CLIP >  = 3.24 were performed using Khi2 or Fisher exact test or Mann Whitney test as appropriate. When a significant difference was noted, spermann R^2^ (coefficient of determination) was performed between CLIP and the considered factor to estimate if the factors were not surrogates of one another. The threshold of 3.24 was selected ad hoc in accordance with a previous paper [8] focusing on aggressive lymphomas in immunocompetent patients.

For the main analysis: univariate and multivariate analyses using Cox models were performed. Four variable were tested at univariate analysis: IPI, TMTV, CLIP and treatment strategy (Rituximab or upfront chemotherapy). Quantitative variables were dichotomized using already published thresholds: IPI ≥ 3 [3], TMTV ≥ 220 cm^3^ [12], CLIP < 3.24 [8]. Multivariate analysis was conducted using a model selection approach. When several factors were collinear, only the factor resulting in the best model based on the Akaike criterion was retained. Derived hazard ratio (HR) and corresponding 95% confidence intervals (95%IC) are reported. Survival data were displayed on Kaplan Meier curves, comparisons were performed using a log-rank test. A *p* value < 0.05 was considered significant.

For the second objective, PET parameters including CLIP were studied both for PFS and OS using the same procedure: an optimal threshold was determined based on survival curves [13]. Relevant factors were then selected using multivariate analysis and model selection.

## Results

### Patients’ characteristics

A total of 97 PTLD patients were identified in the registry (62 male patients, 35 female patients). Transplanted organs were mostly kidney (54 patients, 55.7%), followed by liver (23 patients, 23.7%). The median follow-up time was 3.7 years. The majority of patients had a monomorphic diffuse large B-cell PTLD (76 patients, 78.3%), 12 patients (12.4%) presented a polymorphic PTLD, 5 patients had a monomorphic Hodgkin subtype, 4 patients finally had a Burkitt PTLD. In total 67 patients were treated using a risk-stratified sequential therapy (induction with 4 cycles of rituximab, followed by a treatment depending on the response) as described in the PTLD-1 trial [3] and 30 patients were treated with upfront chemotherapy. Clinical characteristics of the population are summarized in Table 1. Patients’ characteristics were similar except for the proportion of reduction of immunosuppression (70% in the chemotherapy group, 90% in the RSST group), elevated LDH (73% in chemotherapy group) and histology (all 5 Hodgkin lymphomas and all 4 Burkitt lymphomas were in the chemotherapy group).

**Table 1:** Patients characteristics

	Total (n = 97)	RSST (n = 67)	Chemotherapy (n = 30)	Comparison
Clinical data				
Mean age (SD)	54.0 (16.3)	55.1 (16.4)	53.2 (16.1)	0.58
Sex				0.15
Female	35 (36%)	21 (31%)	14 (47%)	
Male	62 (64%)	46 (69%)	16 (53%)	
PS ECOG ≥ 2	27 (28%)	21 (31%)	6 (20%)	0.25
B symptoms (missing: 1)	48 (50%)	33 (49%)	15 (52%)	0.82
Lymphoma characteristics				
Histology				< 0.001*
Monomorphic DLBCL	76 (78%)	56 (84%)	20 (67%)	
Monomorphic HL	5 (5%)	0 (0%)	5 (17%)	
Monomorphic BL	4 (4%)	0 (0%)	4 (13%)	
Polymorphic	12 (12%)	11 (16%)	1 (3%)	
EBER (missing: 2)				0.47
Positive	31 (33)	20 (30%)	11 (38%)	
Negative	64 (67)	46 (70%)	18 (62%)	
Ann Arbor stage				0.81
I	15 (16%)	11 (16%)	4 (13%)	
II	8 (8%)	6 (9%)	2 (7%)	
III	13 (13%)	10 (15%)	3 (10%)	
IV	61 (63%)	40 (60%)	21 (70%)	
Nodal involvement	64 (66%)	41 (61%)	23 (77%)	0.14
Extranodal involvement	77 (79%)	54 (81%)	23 (77%)	0.66
Extranodal organs involved ≥ 2	28 (29%)	18 (27%)	10 (33%)	0.52
Biological results				
Elevated LDH (missing 4)	51 (55%)	32 (48%)	19 (73%)	0.04*
B2m (missing: 41)				
Mean (SD)	5.7 (3.6)	5.7 (3.7)	5.6 (3.5)	0.49
Albumin (missing: 12)				
Mean (SD)	34.1 (5.8)	34.8 (5.7)	32.2 (5.8)	0.05
Prognostic scores				
IPI (Missing: 4)				0.47
0–2	52 (55%)	39(58%)	13 (50%)	
3–5	41 (44%)	28 (42%)	13 (50%)	
Transplantation related data				
Time from transplantation to PTLD (years)				
Mean (SD)	9.4 (7.2)	9.6 (7.6)	9 (6.1)	0.99
Age at transplantation (years)				
Mean (SD)	45.1 (15.9)	45.5 (16.9)	44.2 (13.6)	0.59
Transplant type				0.64
Kidney	54 (56%)	34 (51%)	20 (67%)	
Liver	23 (24%)	17 (25%)	6 (20%)	
Heart	3 (3%)	3 (5%)	0 (0%)	
Lung	2 (2%)	2 (3%)	0 (0%)	
Hematopoietic SCT	8 (8%)	5 (7%)	3 (10%)	
Multiple	7 (7%)	6 (9%)	1 (3%)	
Graft involvement	10 (10%)	6 (9%)	4 (13%)	0.51
Reduction of immunosuppression	79 (81%)	60 (90%)	19 (70%)	0.02*
Baseline PET measurements				
*Mean (SD) [minimum–maximum]*				
Administered activity (MBq/kg)	3.42 (0.90) [1.85–5.01]	3.45 (0.92) [1.85–4.95]	3.38 (0.90) [1.95–5.01]	0.37
CLIP	3.89 (1.10) [1.72–8.9]	3.82 (0.92) [1.98–6.04]	4.06 (1.44) [1.72–8.90]	0.40
TMTV (ml)	250.9 (444.2) [1–3605]	217.5 (476) [1–3603]	325.6 (359.2) [7–1106]	0.12
TLG (ml)	3093 (4931) [9–23718]	2432 (4126) [9–23718]	4570 (6203) [18–21335]	0.09
SUVmax (tumor)	23.7 (13.4) [4.2–66.1]	23.4 (13.2) [4.2–66.1]	24.2 (14.1) [4.3–56.9]	0.79
SUVmean (tumor)	11.5 (6.6) [2.1–32.3]	11.4 (6.6) [2.1–32.3]	11.6 (6.9) [2.6–27.0]	0.90
SUVmax (tumor)/SUVmean (liver)	12.0 (7.2) [2.0–34.3]	11.4 (6.4) [2.0–33.5]	13.4 (8.8) [2.5–34.3]	0.27
SUVmax (tumor)/SUVmean (blood pool)	15.5 (9.3) [2.5–48.25]	14.7 (7.9 [2.5–34.0]	17.2 (11.8) [2.8–48.3]	0.29

*SD* standard deviation, *DLBCL* diffuse large B-cell lymphoma, *HL* Hodgkin lymphoma, *BL* Burkitt lymphoma, *SCT* stem cell transplantation

*p < 0.05

### Prognostic factors: univariate analysis

Among the 89 patients in which CLIP was available (8 missing values corresponding to PET/CT where cerebellum was outside the field of view), 23 had a CLIP inferior to 3.24, resulting in a HR of 2.4 (1.3–4.5) (*p* = 0.005) for PFS and 2.1 (1.0–4.3) (*p* = 0.049) for OS at univariate analysis. Patients with low CLIP were older than those with high CLIP (60.7 vs. 51.7 years, *p* = 0.02), were transplanted later (mean age: 50.5 vs. 43.5, *p* = 0.02) and had lower albumin levels (30.4 g/l vs. 35.0 g/l, *p* = 0.01) (Table 2). R^2^ between CLIP and age remained low (negative correlation with R^2^ = 3.6%), as well as between CLIP and SUVmax (positive correlation with R^2^ = 4.7%). A significantly lower PFS and OS were noted when patients had CLIP < 3.24 (5-year PFS: 19.8% vs. 62.3%, 5-year OS: 36.6% vs. 68.8%).

**Table 2:** Patients characteristics according to CLIP values

	CLIP < 3.24 (n = 23)	CLIP ≥ 3.24 (n = 66)	Comparison
Clinical data			
Mean age (SD)	60.7 (12.9)	51.7 (16.3)	0.02*
Sex			0.44
Female	7 (30.4%)	26 (39.4%)	
Male	16 (69.6%)	40 (60.6%)	
PS ECOG ≥ 2	8 (34.8%)	18 (27.3%)	0.50
B symptoms (missing: 1)	12 (52.2%)	31 (47.7%)	0.71
Lymphoma characteristics			
Histology			0.34
Monomorphic DLBCL	16 (69.6%)	55 (83.3%)	
Monomorphic HL	2 (8.7%)	2 (3.0%)	
Monomorphic BL	1 (4.3%)	2 (3.0%)	
Polymorphic	4 (17.4%)	7 (10.7%)	
EBER (missing: 2)			0.55
Positive	8 (34.8%)	18 (28.1%)	
Negative	15 (65.2%)	46 (71.9%)	
Ann Arbor stage			0.31
I	5 (21.7%)	10 (15.2%)	
II	4 (17.4%)	4 (6.1%)	
III	2 (8.7%)	9 (13.6%)	
IV	12 (52.1%)	43 (65.2%)	
Nodal involvement	17 (73.9%)	42 (63.6%)	0.37
Extranodal involvement	17 (73.9%)	54 (81.8%)	0.55
Extranodal organs involved ≥ 2	7 (30.4%)	18 (27.3%)	0.77
Biological results			
Elevated LDH (missing: 3)	14 (66.6%)	33 (50.7%)	0.22
B2m (missing: 41)			
Mean (SD)	6.9 (4.1)	5.5 (3.5)	0.10
Albumin (missing: 12)			
Mean (SD)	30.4 (6.2)	35.0 (5.5)	0.01*
Prognostic scores			
IPI (Missing: 3)			0.08
0	3 (14.3%)	7 (10.8%)	
1	3 (14.3%)	12 (18.5%)	
2	2 (9.5%)	21 (32.3%)	
3	8 (38.1%)	13 (20.0%)	
4	2 (9.5%)	10 (15.4%)	
5	3 (14.3%)	2 (3.1%)	
Transplantation related data			
Time from transplantation to PTLD (years)			
Mean (SD)	10.8 (8.9)	8.7 (6.4)	0.09
Age at transplantation (years)			
Mean (SD)	50.5 (13.1)	43.5 (16.3)	0.02*
Transplant type			0.45
Kidney	14 (60.8%)	33 (50.0%)	
Liver	7 (30.4%)	16 (24.2%)	
Heart	0 (0.0%)	3 (4.5%)	
Lung	1 (4.3%)	1 (1.5%)	
Hematopoietic SCT	0 (0.0%)	7 (10.6%)	
Multiple	0 (0.0%)	6 (9.1%)	
Graft involvement	3 (13.0%)	6 (9.1%)	0.69
Reduction of immunosuppression	21 (91.3%)	51 (81.0%)	0.33
Rituximab alone first	16 (69.6%)	46 (69.7%)	0.99
Baseline PET measurements			
*Mean (SD) [minimum–maximum]*			
Administered activity (MBq/kg)	3.81 (0.94) [1.95–4.95]	3.45 (0.89) [1.85–5.01]	0.06
TMTV (ml)	350.3 (752.1) [20–3603]	224.1 (298.7) [1–1106]	0.37
TLG (ml)	3245.6 (5564) [101–23718]	3271.2 (4986.5) [9–21335]	0.98
SUVmax (tumor)	19.2 (8.28) [6.0–37.6]	25.3 (15.0) [4.2–66.1]	0.02*
SUVmean (tumor)	9.9 (4.1) [4.5–20.5]	12.2 (7.4) [2.1–32.3]	0.06
SUVmax (tumor)/SUVmean (liver)	9.4 (4.6) [3.5–21.0]	13.0 (8.0) [2.0–34.3]	0.01*
SUVmax (tumor)/SUVmean (blood pool)	12.4 (6.1) [4.3–28.3]	16.7 (10.4) [2.4–48.2]	0.02*

*p < 0.05

An IPI score of more than 3 was identified in 41 patients (4 patients had missing data). IPI was a significant predictor of both PFS and OS at univariate analysis (HR 2.3 in both cases, *p* < 0.05). Among its item, only PS ECOG ≥ 2 reached significance for PFS. Derived 5-year PFS were 39.1% (IPI ≥ 3) versus 60.8% (IPI < 3) and derived 5-year OS were 51.4% (IPI ≥ 3) vs 69.2% (IPI < 3).

**Fig. 1:**
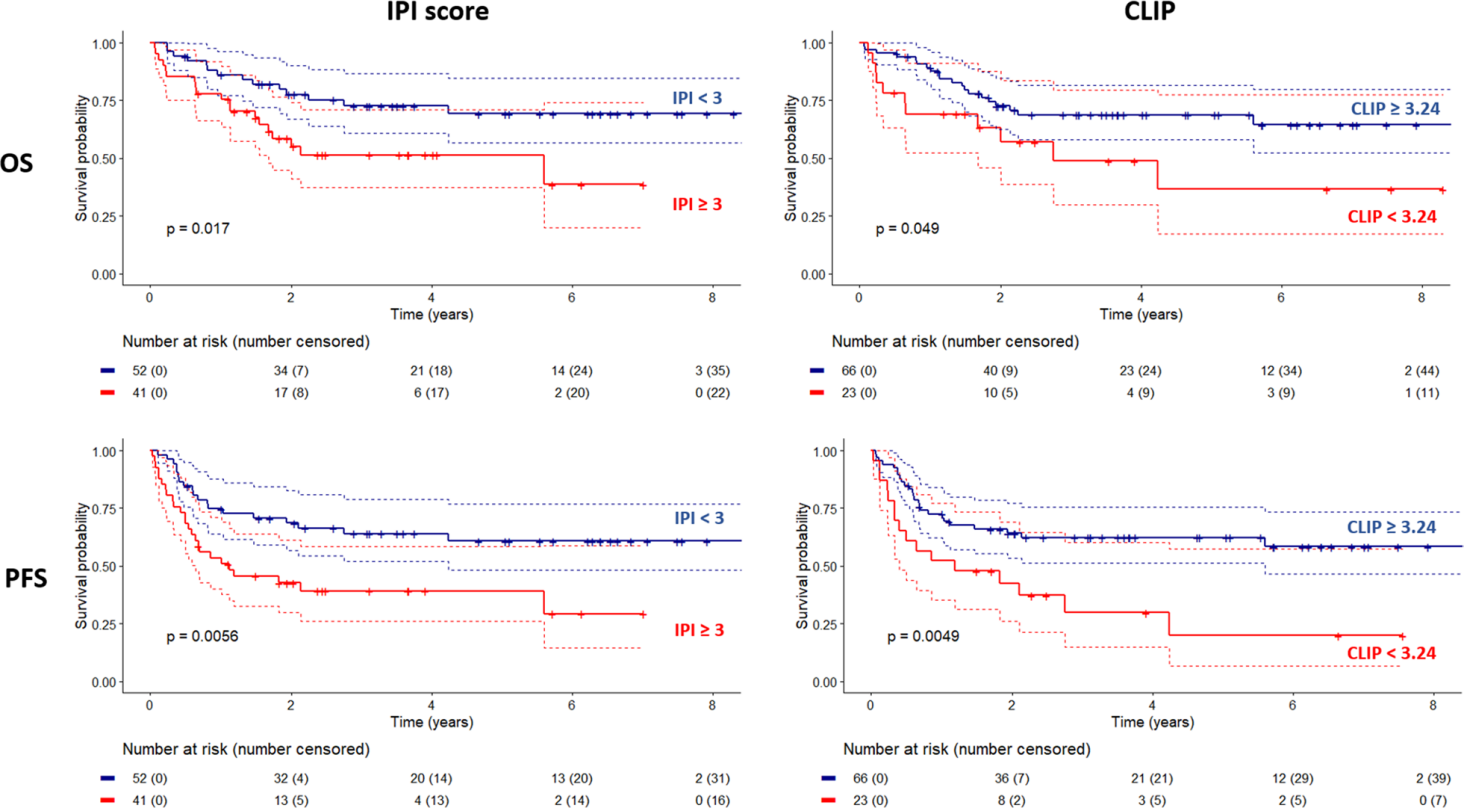
Survival curves based on IPI score and cerebellum/liver index (CLIP). *OS* overall survival, *PFS* progression free survival

TMTV, using a threshold of 220 cm^3^ was not a significant risk factor of neither PFS or OS. Treatment strategy was not a prognostic factor of PFS or OS. The results are presented in Table 3 and Fig. 1.

**Table 3:** Uni and multivariate analyses

	% at risk (# of unavailable data)	Univariate analysis (log-rank)	Multivariate analysis (cox models)
PFS			
IPI score (≥ 3)	44.1% (NA:4)	HR 2.3 [1.3–4.2] *p* = 0.006*	HR 2.0 [1.0–4.0] *p* = 0.040*
CLIP (< 3.24)	25.8% (NA:8)	HR 2.4 [1.3–4.5] *p* = 0.005*	HR 2.4 [1.3–4.5] *p* = 0.008*
TMTV (≥ 220 ml)	30.9%	HR 1.3 [0.7–2.5] *p* = 0.388	HR 0.9 [0.5–1.8] *p* = 0.746
Rituximab treatment	61.1%	HR 1.0 [0.5–1.9] *p* = 0.992	–
Reduction of IS	81.4%	HR 0.6 [0.3–1.3] *p* = 0.224	–
Sex (male)	64.0%	HR 1.3 [0.7–2.5] *p* = 0.369	–
B symptoms	50.0% (NA:1)	HR 1.4 [0.7–2.5] *p* = 0.267	–
Age > 60	41.2%	HR 1.4 [0.8–2.4] *p* = 0.323	–
Ann Arbor stage ≥ 3	76.0%	HR 0.8 [0.4–1.6] *p* = 0.620	–
Elevated LDH	55.0% (NA:4)	HR 1.4 [0.8–2.6] *p* = 0.230	–
PS ECOG ≥ 2	28.0%	HR 1.9 [1.0–3.4] *p* = 0.050*	–
Extranodal organs involved ≥ 2	29.0%	HR 1.0 [0.5–2.0] *p* = 0.934	–
OS			
IPI score (≥ 3)	44.1% (NA:4)	HR 2.3 [1.1–4.6] *p* = 0.017*	HR 1.7 [0.8–3.9] *p* = 0.171
CLIP (< 3.24)	25.8% (NA:8)	HR 2.1 [1.0–4.3] *p* = 0.049*	HR 2.1 [1.0–4.4] *p* = 0.059
TMTV (≥ 220 ml)	30.9%	HR 1.6 [0.8–3.3] *p* = 0.169	HR 1.1 [0.5–2.5] *p* = 0.757
Rituximab treatment	61.1%	HR 0.9 [0.4–1.8] *p* = 0.655	–
Reduction of IS	81.4%	HR 0.6 [0.3–1.6] *p* = 0.347	–
Sex (male)	64.0%	HR 1.0 [0.4–2.3] *p* = 0.963	–
B symptoms	50.0% (NA:1)	HR 2.0 [0.8–4.6] *p* = 0.123	–
Age > 60	41.2%	HR 0.8 [0.3–1.8] *p* = 0.566	–
Ann Arbor stage ≥ 3	76.0%	HR 0.8 [0.3–1.9] *p* = 0.626	–
Elevated LDH	55.0% (NA:4)	HR 1.9 [0.8–4.4] *p* = 0.132	–
PS ECOG ≥ 2	28.0%	HR 2.2 [1.0–4.8] *p* = 0.065	–
Extranodal organs involved ≥ 2	29.0%	HR 1.9 [0.9–4.3] *p* = 0.117	–

For univariate analysis, the *p* value displayed correspond to a log-rank test. Confidence intervals and multivariate analyses are based on Cox models

*p < 0.05

### Prognostic factors: multivariate analysis

For PFS, both IPI score (≥ 3) and CLIP (< 3.24) HR were significant: 2.0 (1.0–4.0) (*p* = 0.04) and 2.4 (1.3–4.5) (*p* = 0.008) respectively. For OS, CLIP resulted in a HR of 2.6 (*p* = 0.059). Neither IPI score or TMTV reached significance.

### Exploratory analysis

Optimal threshold for several PET parameters are presented in Table 4. CLIP optimal threshold was 2.605, lower than the one used in the main analysis and was significantly predictive of PFS ans OS at multivariate analysis (HR of 4.18 and 4.03 respectively). TMTV optimal threshold was higher than 220 ml (423.5 ml for PFS and 331 ml for OS). Neither TMTV or TLG reached significance at multivariate analysis. Tumoral SUVmax, SUVmean as well as ratios (tumor/liver, tumor/blood pool) were not predictors of PFS. However, using their optimal cut-off, SUVmean, Tumor/liver ratio and tumor/blood pool ratio were significants when analyzed separately. Only tumor/blood pool ratio reached significance for OS prediction at multivariate.

**Table 4:** Exploratory analysis on PET parameters

	Optimal threshold	% at risk (# of unavailable data)	Log-rank analysis at optimal threshold	Multivariate analysis with model selection (cox models)
PFS				
CLIP	< 2.605	11.2% (NA:8)	HR 3.70 [1.66–7.69] *p* < 0.001*	HR 4.18 [1.874–9.356] *p* < 0.001*
TLG	> 7830 ml	13%	HR 2.35 [1.12–4.97] *p* = 0.021*	Not selected
TMTV	> 423.5 ml	21.6%	HR 2.0 [1.05–3.85] *p* = 0.033*	Not selected
SUVmax	> 34.52	17.5%	HR 1.74 [0.88–3.46] *p* = 0.11	Not selected
SUVmean	> 4.335	88.7%	HR 2.46 [0.76–8.04] *p* = 0.12	Not selected
Tumor/liver	> 17.58	18.6%	HR 1.74 [0.88–3.46] *p* = 0.11	HR 1.919 [0.92–4.00] *p* = 0.082
Tumor/blood pool	> 16.51	36.1%	HR 1.77 [0.97–3.21] *p* = 0.058	Not selected
OS				
CLIP	< 2.605	11.2% (NA:8)	HR 3.84 [1.39–10.0] *p* = 0.005*	HR 4.03 [1.27–12.8] *p* = 0.018*
TLG	> 9191 ml	10.3%	HR 3.95 [1.57–9.96] *p* = 0.017*	HR 1.83 [0.64–5.26] *p* = 0.263
TMTV	> 331 ml	23.7%	HR 3.53 [1.58–7.9] *p* = 0.0011*	Not selected
SUVmax	> 26.19	36.1%	HR 2.15 [0.96–4.81] *p* = 0.056	Not selected
SUVmean	> 14.39	27.8%	HR 2.36 [1.05–5.27] *p* = 0.031*	Not selected
Tumor/liver	> 10.83	47.4%	HR 3.53 [1.4–8.91] *p* = 0.0043*	Not selected
Tumor/blood pool	> 14.8	44.3%	HR 4.44 [1.76–11.21] *p* < 0.001*	HR 3.41 [1.06–10.96] *p* = 0.039*

*p < 0.05

## Discussion

Our study demonstrates that the cerebellum/liver index is an independent predictor of PFS in PTLD. For OS, it was at the edge of significance (*p* = 0.059) although significant on univariate analysis (*p* = 0.049). Using the cut-off already published in the literature at 3.24 [8], patients with a low CLIP have an approximately two fold increased risk of progression. This excess risk is similar to what has been observed in immunocompetent diffuse large cell B-cell lymphoma [8]. Our exploratory analysis of PET/CT parameters shows that significance would have been achieved for both PFS and OS by choosing a lower CLIP threshold (2.605). However, we chose to use a previously published threshold to avoid overfitting bias.

This ratio has the advantage of being reproducible and easily measurable, provided that the skull is integrated in the PET field of view [7, 8].

The mechanisms underlying this index remain poorly understood: a metabolic theft of lymphoma cells at the expense of cells from healthy organs is often considered [8, 14]. An inverse correlation is sometimes shown between healthy organ uptake and TMTV [7, 8, 14, 15] but tumor volume is probably not the only determinant. CLIP seems to partly integrate age and albumin: patients with low CLIP were indeed significantly older and had lower albumin levels.

Albuminemia was not included because of a large number of missing data. Age, although a slight negative correlation is noted, explains only 3.6% of variability of the CLIP value.

Glycemia was not available. Higher glucose levels are reported to lower cerebral and hepatic uptake levels [16]. The use of a ratio between the two should have helped to mitigate this effect.

The IPI score, with a cutoff of 3, is confirmed as an independent prognostic factor for PFS. However, it did not reach significance for OS prediction when studied in conjunction with CLIP, factor that was not included in the previously published studies [1, 3, 4].

The treatment of PTLD is heterogeneous and relies primarily on the reduction of immunosuppressive treatments. While PTLD was initially treated as their de novo counterpart, using CHOP chemotherapy, a more conservative approach can now be used [3, 17]. This approach consists of treating the patient with 4 weekly cycles of rituximab followed by either rituximab maintenance or (R)-CHOP chemotherapy depending on the response to induction. Our population is thus heterogeneous in terms of treatment: 67/97 patients received an RSST strategy and 30/97 received chemotherapy upfront. However, the choice of treatment had no impact in terms of survival (PFS or OS) as we could verify (Table 3).

TMTV did not appear to be significantly associated with survival in either univariate or multivariate studies. We used a threshold of 220 ml, which has been reported to be prognostic for large B-cell lymphomas in immunocompetent patients [12]. Our exploratory analysis shows that a higher cut-off would have led to better results (423.5 ml for PFS and 331 ml for OS). However TMTV in this favorable setting still did not reached significance at multivariate analysis.

The limitations of our study are related to its retrospective design. PTLDs remain indeed rare lymphomas making any prospective collection difficult. In particular, PET scans were acquired in several centers with different administered activity, ranging from 1.85 to 5.01 MBq/kg, leading to probable variations in SUV estimation with higher cerebellar SUVmax on newer systems. However, the use of a ratio allows to limit the consequences of this effect. The histological subtype was also heterogeneous in our population with, however, a majority of diffuse monomorphic B large cell PTLD (78.3%). The number of patients did not allow for a subgroup analysis.

## Conclusion

The cerebellar liver index is a promising predictor of progression-free survival and perhaps overall survival in PTLD. Further prospective studies are needed to confirm these results.

## Data Availability

The datasets generated during the current study are not publicly available due to data protection policies but are available from the corresponding author on reasonable request.

## Abbreviations

18F-FDG: 18F-fluorodeoxyglucose
CLIP: Cerebellum-to-liver uptake ratio for prognostis
EBER: Epstein Barr virus status of the tumor
ECOG PS: Eastern cooperative oncology group performans status
HR: Hazard ratio
IPI: International prognostic index
OS: Overall survival
PET/CT: Positron emission tomography computed tomography
PFS: Progression-free survival
PTLD: Post-transplant lymphoproliferative disorders
ROI: Region of interest
SUV: Standard uptake value
TMTV: Total metabolic tumor volume
